# Genetic Engineering of Natural Killer Cells for Enhanced Antitumor Function

**DOI:** 10.3389/fimmu.2020.607131

**Published:** 2020-12-16

**Authors:** Simone Mantesso, Dirk Geerts, Jan Spanholtz, Lucia Kučerová

**Affiliations:** Research and Development, Glycostem Therapeutics, Oss, Netherlands

**Keywords:** natural killer cells, tumor, genetic engineering, transduction, transfection, chimeric antigen receptor-natural killer cells, activating receptors, inhibitory receptors

## Abstract

Natural Killer (NK) cells are unique immune cells capable of efficient killing of infected and transformed cells. Indeed, NK cell-based therapies induced response against hematological malignancies in the absence of adverse toxicity in clinical trials. Nevertheless, adoptive NK cell therapies are reported to have exhibited poor outcome against many solid tumors. This can be mainly attributed to limited infiltration of NK cells into solid tumors, downregulation of target antigens on the tumor cells, or suppression by the chemokines and secreted factors present within the tumor microenvironment. Several methods for genetic engineering of NK cells were established and consistently improved over the last decade, leading to the generation of novel NK cell products with enhanced anti-tumor activity and improved tumor homing. New generations of engineered NK cells are developed to better target refractory tumors and/or to overcome inhibitory tumor microenvironment. This review summarizes recent improvements in approaches to NK cell genetic engineering and strategies implemented to enhance NK cell effector functions.

## Introduction 

**Figure 1:**
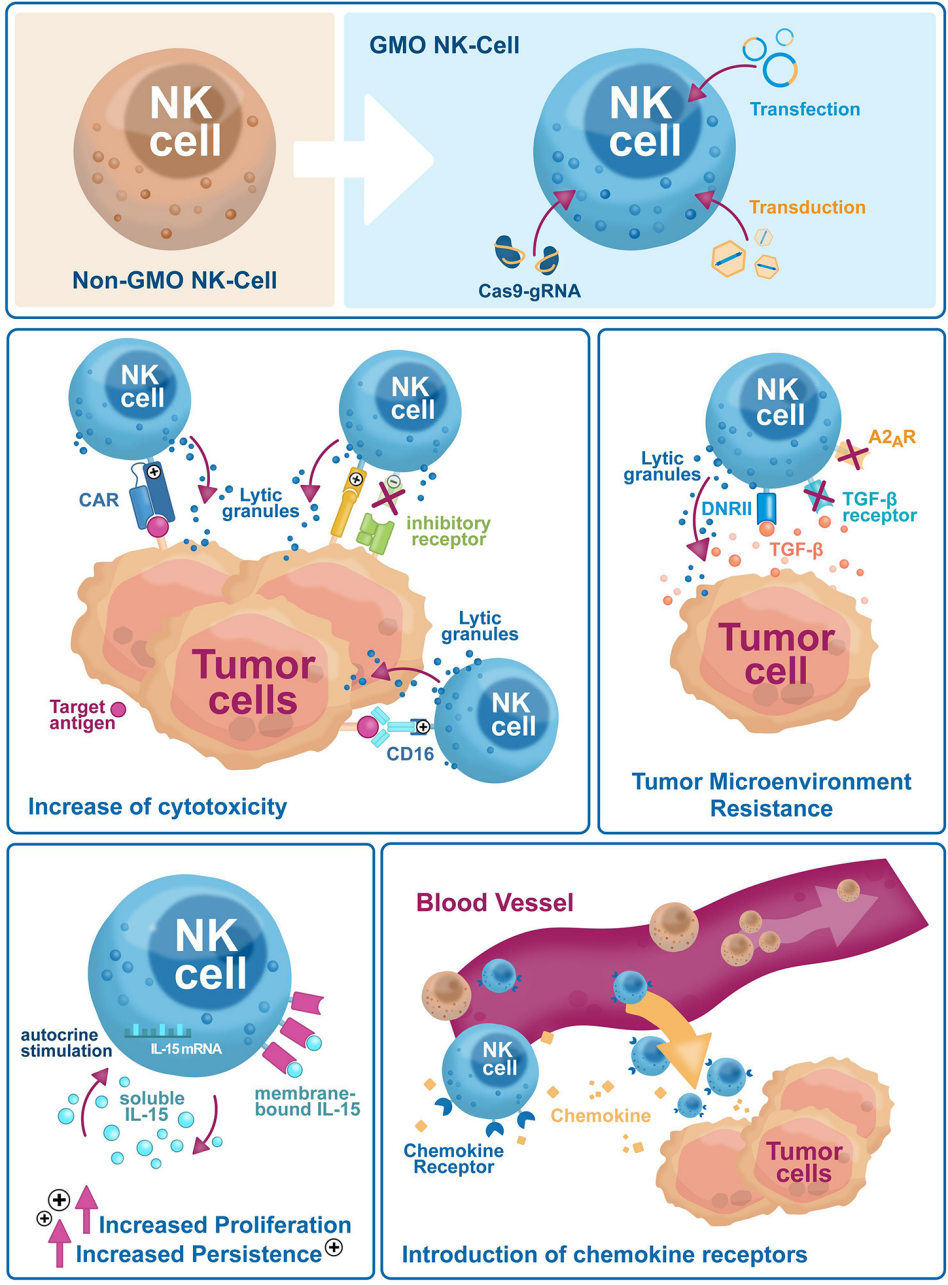
Genetic engineering has broad applications to enhance NK cell-based immunotherapies efficacy against tumor escape mechanisms. Genetic engineering is used on NK cells to improve their cytotoxicity (i.e. exogenous expression of CARs and activating receptors, or selective downregulation of inhibitory receptors), reduce sensitivity to the tumor microenvironment (i.e. downregulation of inhibitory cytokine and small molecule receptors, the introduction of dominant-negative receptors), increase *in vivo* proliferation and persistence via autocrine cytokines stimulation and tumor homing (i.e. expression of chemokine receptors).

Natural killer (NK) cells are part of the innate immune system. Discovered more than 40 years ago, they kill virus-infected cells, counteract tumor formation and initiate innate immune responses (1). Lower NK cell counts and reduced cytotoxicity are associated with higher cancer risks (2, 3), as NK cells kill aberrant somatic cells with downregulated major histocompatibility complex class I (MHC-I) molecules that escape T-cell scrutiny (1). Immunotherapy is a powerful biological therapy for boosting the patient’s immune system, helping it to fight cancer off. Currently, immunotherapy options include compounds like monoclonal antibodies, cancer vaccines, and checkpoint inhibitors, and more recently cellular products like T cells, dendritic cells or NK cells. Early results showed that NK cells can be a safer alternative over T cells due to reduced side effects. Currently however, NK cell *ex vivo* expansion technologies are laborious, and their persistence *in vivo* is limited. Genetic engineering is a valuable tool to overcome these limitations and improve NK cells target specificity and cytotoxicity. NK cells were difficult to genetically modify but recently, NK cell engineering has become efficient and reproducible. This review will summarize recent improvements of NK cell engineering and discuss their use in increasing antitumor efficacy and *in vivo* persistence through improved tumor homing, and higher target specificity and cytotoxicity (**Figure 1**).

## Functionality and Mechanism of Action of Natural Killer Cells

NK cells represent the main innate lymphocyte cell type. They mediate both anti-tumor and anti-viral responses. Only anti-tumor effects will be the subject of this review. NK cells are generally classified as CD56^+^CD3^-^ lymphoid cells and further subdivided into two major subpopulations based on CD56 and CD16 receptor surface expression: CD56^dim^CD16^bright^ and CD56^bright^CD16^dim^ cells (4, 5). Circulating CD56^dim^CD16^bright^ NK cells are quiescent but become highly cytotoxic upon recognition of target cells, CD56^bright^CD16^dim^ cells, that reside in secondary lymphoid tissues, constitutively produce cytokines (6, 7). NK cells killing ability is tightly regulated by a wide range of inhibitory and activating receptors (1). The most prominent inhibitory receptors are the inhibitory killer-cell immunoglobulin-like receptors (KIRs), that bind polymorphic classical MHC-I molecules (HLA-ABC), universally expressed on healthy cells (8). A major role is also played by the inhibitory heterodimer receptor CD94-NKG2A that binds the non-classical MHC-I molecule HLA-E (9, 10). NK cell activating receptors comprise DNAM-1, NKG2D, CD94/NKG2C, CD94/NKG2E, natural cytotoxicity receptors (NCRs) like NKp30, NKp44, NKp46, and CD16 (11–14) and activating KIRs. All these recognize specific ligands on the surface of target cells: the CD94/NKG2C and CD94/NKG2E heterodimers recognize HLA-E molecules, the NKG2D receptor recognizes the MHC class-I-chain related proteins A and B (MICA and MICB) and UL16 binding proteins (ULBPs), and DNAM-1 recognizes nectin-2 (CD112) and nectin-like proteins (15, 16). Upon activation, NK cells release lytic granules containing perforin and granzyme B within the immunological synapse to kill target cells (17). NK cells can also exert antibody-dependent cell cytotoxicity (ADCC) by recognizing antibody-coated cells through the low-affinity receptor for the Fc portion of IgG_1_ antibodies (FcγRIIIa or CD16). Activated NK cells also secrete soluble factors like tumor necrosis factor α (TNF-α), TNF-related apoptosis-inducing ligand (TRAIL) and Fas ligand (FasL) to trigger apoptosis in target cells (18). And finally, they secrete interferon γ (IFN-γ) (19), growth factors (GM-CSF), immunoregulatory cytokines (IL-15, IL-10, and IL-13) and chemokines (20, 21). These cytokines modulate both innate and adaptive immune responses, such as dendritic cell (DC) maturation and CD4^+^ to Th1 T cell differentiation, respectively (22–24).

## Tumor Cells Escape NK Cells Surveillance

NK cells prevent tumor formation and metastases (25). Blood NK cell counts positively correlate with lower risk for cancer development (2), whereas higher tumor tissue NK cell infiltration correlates with improved treatment outcomes (26, 27). This antitumor effect has been comprehensively summarized elsewhere recently (21). However, solid tumors develop escape mechanisms to avoid NK cell recognition (28). They can upregulate MHC-I expression and thereby engage inhibitory NK cell receptors. For example, HLA-E upregulation increases engagement of the NKG2A/CD94 heterodimer in IFN-γ-stimulated ovarian cancer cell lines, dampening NK cell activity (29). Another resistance mechanism involves the downregulation or shedding of NK cell activating receptor ligands (30). Recently, it was found that NK cells express the immune checkpoint inhibitor PD-1 and that their cytotoxic activity is reduced upon PD-1 engagement in PD-L1 expressing tumors (31). Another recognized key player for tumor cell survival and immune system escape is the tumor microenvironment (TME). The TME comprises tumor-associated non-malignant cells and extracellular matrix components. It secretes suppressive cytokines, like transforming growth factor (TGF)-β (32) and IL-10 (33), or suppressive factors like prostaglandin E2 (PGE2) and adenosine (34, 35) that prevent NK cell mobilization and target tissue infiltration. Most of these immunosuppressive factors are secreted by tumor-associated cells, mainly regulatory T cells (T-regs) (36), M2 macrophages and myeloid-derived suppressor cells (MDSCs) (37). Additionally, the TME can be hypoxic by the tumor’s high metabolism and poor vascularization, impairing NK cells cytotoxic activity (38). In conjunction, hypoxia favors the selection of the phenotypically most aggressive clones, and tumors become capable of sustained proliferation and metastatic potential that can no longer be controlled by NK cell action (39). Therefore, novel approaches for sustaining NK cells antitumor action are crucial for the development of effective tumor therapy.

## NK Cell Genetic Modification for Sustained Functionality in Cancer Immunotherapy

Several clinical trials have confirmed the safety profile and efficacy of adoptive NK cells as treatment for hematological malignancies (21). NK cells do not mediate severe toxicities like graft-versus host disease (GvHD) or cytokine release syndrome (CRS) and, therefore, do not require stringent HLA matching (40, 41), the bane of T cell therapies. Consequently, NK cells derived from a single donor can be used to treat several patients. This “off-the-shelf application” dramatically improves therapy access and reduces production times and costs. The trials however also highlighted a relatively limited effectivity against solid tumors (21). This is caused by low tumor homing and infiltration, short *in vivo* persistence and impaired NK cell activity in the cancer patients, this by tumor antigen downregulation and the immunosuppressive TME (28). To overcome these drawbacks, genetic modifications of NK cells has been suggested, and will be discussed in detail in this study. Resting primary NK cells from peripheral blood (PB) or umbilical cord blood (UCB) are difficult to engineer by commonly used approaches, like lentivirus, as low transduction efficiencies were always reported (42). Reduced transduction efficiency rates may be explained by the strong antiviral mechanisms NK cells possess (43). Lentiviral transduction can activate innate immune receptor signaling and trigger NK cell apoptosis. Retrovirus showed higher transduction rates in NK cell lines (especially NK-92) or *ex vivo* activated and expanded NK cells, mostly PB-NK cells (44) (**Table 1**). Standard retroviral transduction methods (γ-retroviruses), though not known to alter NK cell phenotype and function (45), carry the risk of insertional mutagenesis due to their preference of inserting into active gene promoters, and thereby represent a yet unresolved safety concern, especially if high multiplicity of infection (MOI) rates are used. Besides, NK cell viability after retroviral transduction has been seldom reported and data are scarce. On the other hand, lentiviral transduction methods do not require actively dividing cells, conversely to standard retroviral systems (57). All viral systems are limited in insert size (< 10 kb). All in all, transduction efficiencies remain variable, depending on the NK cell source and MOI (which is seldom reported), and sufficient transduction may require either multiple transductions rounds or post-transduction cell enrichment (**Table 1**). Alternatively, high transgene expression levels in both primary and *ex vivo* expanded NK cells (58, 59), can be achieved with transfection methods like electroporation and lipofection, that are not limited in insert size (**Table 2**). Transgene expression after transfection is however not stable over time, strongly reducing its appeal for clinical applications. Other non-viral genetic integration systems like transposons exist with the advantage to not be limited in insert size. Sleeping Beauty and PiggyBac are the most common and well-characterized transposon systems but have so far mainly been tested in T cells, and NK cell studies are still rare and lack safety data. Alternatively, the CRISPR/Cas9 system has been extensively investigated for targeted engineering, with already some studies in NK cells (65). Although CRISPR/Cas9 can be delivered by both viral and non-viral systems, non-viral delivery of a ribonuclear protein (RNP) complex made up by the Cas9 nuclease and the single guide RNA (sgRNA) is preferred, since it limits off-target effects due to viral DNA integration. However, *ex vivo* non-viral delivery requires optimization, as efficiency is often very limited and viability a concern (61). Of note, CRISPR/Cas9 can be used to efficiently screen effective synthetic constructs electroporated into T cells (66), significantly speeding up the discovery of constructs for reprogramming adoptive NK cell functionality and specificity.

**Table 1:** Genetic modification of NK cells with viral methods.

NK source	Pre-stimulation	Method	Envelope	Construct	Target(s)	MOI	Efficiency	Reference
**PB**	Co-culture with K562-mbIL15-41BBL and IL-2 10 IU/ml	Retrovirus	RD114	αCD19-BB- CD3ζ CAR	CD19	n.r.	43%–93%	(44)
**PB**	Co-culture with K562-mbIL15-41BBL	Retrovirus	RD114	αCD19-2B4- CD3ζ CAR	CD19	n.r	24% ± 7.4%	(45)
**PB**	Co-culture with K562-mbIL15-41BBL and IL-2 10 IU/ml	Retrovirus	RD114	Membrane-bound IL-15	none	n.r.	40%–63%	(46)
**PB**	Co-culture with K562-mbIL15-41BBL and IL-2 100 IU/ml	Retrovirus	RD114	hTERT	none	n.r	51%–65%	(47)
**PB**	Co-culture with EBV-LVL	Retrovirus	gp100-specific T cell receptor	NGFR	none	n.r.	26%–93%	(48)
				CXCR2	CXCL1-2-6-8			
**PB**	Co-culture with K562-mbIL15-41BBL and IL-2 10 IU/ml	Retrovirus	Murine stem cell virus	NKG2D-DAP10- CD3ζ	MICA, MICB, ULPB	n.r.	28%	(49)
**PB**	Co-culture with K562-mbIL15-41BBL and IL-2 400 IU/ml	Retrovirus	Murine stem cell virus	Anti-NG2A PEBL	NKG2A	n.r.	49%–82%	(50)
**UCB**	IL-15 10 ng/ml	Retrovirus	RD114	DNRII	TGF-β	n.r.	61%–88%	(51)
**UCB**	Co-culture with K562-mbIL15-41BBL and IL-2 200 IU/ml, IL-15 15 ng/ml	Retrovirus	n.r.	Dominant negative TGF-β receptor (NKA)	TGF-β	n.r.	43% ± 27%	(52)
**PB**	IL-12 50 ng/ml	Lentivirus	VSV-G	αNKG2A shRNA and eGFP	NKG2A	10	50% ± 20%	(53)
**PB**	IL-15 10 ng/ml	Lentivirus	VSV-G	αCD19-CD28-CD3ζ CAR	CD19	5–10	0%–34 %	(42)
		α-retrovirus	RD114-TR	αCD19-CD28-CD3ζ CAR	CD19	10	48%	
		lentivirus					41%	
**PB**	IL-2 500 U/ml IL-15 10 ng/ml	Lentivirus	BaEV	αCD19-4-1BB-CD3ζ CAR	CD19	10	70%	(54)
			VSV-G	eGFP	none	0.1–10	<3%	
**CD34+ HSC from UCB**	Quiescent	Lentivirus	VSV-G	eGFP	None	10	0%	(55)
			RD114/TR				< 5%	
			BaEV				15%–30%	
	SCF/TPO/Flt-3l		VSV-G	eGFP	None	10	< 5%	
			RD114/TR				30%–50%	
			BaEV				80%–90%	
**NK-92**	IL-2 50 IU/ml	Lentivirus	VSV-G	IL-15 and eGFP	None	n.r.	4% (eGFP)	(56)

Studies based on NK cells lines other than NK-92 have not been reported in this table, as they have not been employed in clinical trials yet. Studies where the transduction efficiency was not reported, or post-transduction enrichment/selection was performed without mentioning the original transduction efficiency were excluded. RD144, serotype of endogenous feline type C virus; n.r., not reported; DNRII, dominant negative TGF-β RII; NGFR, Nerve Growth Factor Receptor; PEBL, protein expression blocker.

**Table 2:** Genetic modification of NK cells with non-viral methods.

NK type/source	Pre-stimulation	Method	Construct	Targets	Efficiency	Reference
**PB**	Non-expanded	Electroporation	αCD19-4-1BB-CD3ζ CAR	CD19	18%–59%	(58)
	Co-culture with K562-mbIL15-41BBL and IL-2 100 IU/ml (1000 IU/ml 24h before electroporation)				28%–92%	
**PB**	IL-2 600 U/ml	Electroporation	αCD19-4-1BB-CD3ζ CAR	CD19	40%	(59)
			CCR7	CCL19-CCL21	20%	
**PB**	Co-culture with EBV–SMI–LCL and IL-2 500 IU/ml	Electroporation	CD34	none	> 80%	(60)
			CCR7 and CD16-158V	CCL19-CCL21 and Fc receptors	n.r.	
**NK-92**	IL-2 100 IU/ml	Cas9 RNP nucleofection	SFFV promoter	CD16	1.2 %	(61)
				DNAM-1	3.5%	
**iPSCs**	Maintained in Matrigel-coated plate for 5 days before nucleofection	Nucleofection	αMesothelin- 2B4-CD3ζ CAR	Mesothelin (ovarian cancer)	n.r.	(62)
**NK-92**	IL-2 1000 U/ml	Nucleofection	DNTβRII	TGF-β	20% ± 2.5%	(63)
**NK-92**	IL-2 400 IU/ml	Not specified	NKG2D-DAP10- CD3ζ CAR	MICA, MICB, ULPB	n.r.	(64)

N.r., not reported; DNTβRII, dominant negative TGF-β RII.

Until recently, these limitations delayed NK cell genetic reprogramming for large scale applications compared to T-cells reprogramming. Novel transduction enhancers (e.g. RetroNectin and vectofusin-1) and studies on alternative viral envelopes, mostly genetically modified versions of baboon endogenous retrovirus (BaEV) and RD114 feline retrovirus glycoprotein (42, 54, 55), contributed to improved NK cell transduction efficiency, rekindling the interest in genetic manipulation of NK cells (**Table 1**). The BaEV envelope consistently displayed efficient NK cell transduction and might replace the standard VSV-G envelope in NK cell applications. NK cells can efficiently be differentiated from CD34+ hematopoietic stem and progenitor cells (HSPCs) (67) derived from UCB, human embryonic stem cells (hESCs), or induced pluripotent stem cells (iPSCs) (68, 69). Both CD34+ HSPCs and iPSCs can be effectively modified with VSV-G, RD114, and BaEV-pseudotyped lentiviruses and then differentiated into mature NK cells, and offer another attractive source to generate modified NK cells (55).

In summary, since integrative genetic modification systems are preferred in clinical settings, retrovirus-based genetic engineering has been the established platform to modify NK cells so far. The γ-retroviral systems are being gradually overtaken by lentiviruses after the discovery of new NK cell-specific envelopes and are set to be the mainstay in NK cell engineering for several years. Studies implementing non-viral strategies have emerged in the last years as well, although they are as of yet mostly limited to pre-clinical settings (**Figure 1**).

## Strategies to Increase Tumor-Specific NK Cell Cytotoxicity

### NK Cells Expressing Chimeric Antigen Receptor

Chimeric antigen receptors (CARs) are antibody-based receptors designed to recognize specific ligands on the surface of target cells. All CAR constructs contain an extracellular antibody single-chain variable fragment (scFvs) fused to a transmembrane region and intracellular immune cell activation domains. The first-generation CAR contained only the intracellular CD3ζ stimulatory domain of the T cell receptor (TCR) as activation domain. The second and third generations include one or two additional co-stimulatory domains, respectively (e.g. 4-1BB and/or CD28). CAR technology was first applied on T cells and although CAR-T cells exhibited strong antitumoral clinical responses, their clinical application is severely curtailed by severe toxicities (GvHD and CRS). This allows only autologous applications for CAR-T cells, which decreases the speed of intervention (negatively affecting therapeutic outcomes), increases production costs and thus decreases treatment accessibility. In addition, T cells for autologous use are necessarily derived from heavily pre-treated patients, which impinges on their functionality. CAR-NK cells, however, can be derived from allogeneic sources, without apparently causing neither GvHD nor CRS in the recipient, potentially related to their short *in vivo* persistence and lack of clonal expansion. Off-the-shelf CAR-NK cells can, therefore, have a huge advantage in terms of manufacturing time, costs and accessibility. Besides, allogeneic NK cells are derived from healthy patients and thus retain their normal activity. This allows CAR-NK cells to still exert their anti-tumoral effect in case the CAR expression is decreased or lost, in contrast to CAR-T cells. Only a few CAR-NK phase I/II trials have been started in the last years. Most registered clinical trials employ the NK-92 NK cell line, and the CAR constructs used were based on targets developed for CAR-T cells. This knowledge gap is largely caused by the huge success of anti-CD19 CAR-T cells and the vast amount of clinical data available from CAR-T cell therapies, and as of yet precludes critical assessment of clinal CAR-NK applications. Currently, two CAR-NK cell trials targeting CD19 for leukemia treatment are ongoing. The first one is at the MD Anderson Cancer Centre of the University of Texas (NCT03056339) and is based on UCB-NK cells transduced with a CD19-targeting scFv, interleukin 15 (to enhance NK cells persistence) and an inducible caspase-9 (iC9) suicide gene as a failsafe mechanism. Preliminary results demonstrated that the approach is safe, despite only partial HLA matching between donor and recipient, and potency is high (seven out of 11 patients achieved complete remission) (70). Of note, CAR.19/IL-15/iC9-NK cells were detected at low levels up to 12 months after the beginning of the treatment, whereas they normally disappear within 2 weeks. Unfortunately, no data about exhaustion is reported, and the influence of pre-conditioning treatment could not be established. The second trial (NCT02892695) is led by PersonGen BioTherapeutics (Suzhou, China), and is based on the NK-92 cell line transduced with a third-generation CAR (4-1BBL-CD28-CD3ζ co-stimulatory domains). A third phase I study targeting CD19 with haplo-identical PBNK cells for B-ALL treatment has been completed (NCT00995137), but no results are available so far. The NK-92 cell line is employed in most of the other phase I/II studies, targeting CD7 (NCT02742727) for lymphoma and leukemia, CD33 for the treatment of Acute Myeloid Leukemia (NCT02944162), HER2 against glioblastoma (NCT03383978) and Mucin-1 (MUC1) in MUC1-positive relapsed or refractory solid tumor-like colorectal carcinoma (CRC) and gastric carcinoma. These and other trials are listed and summarized in **Table 3**. Efforts directed against multiple myeloma, with CD138 (71) and SLAMF7 (72) as main targets, are still in the preclinical phase. CAR-NK cells targeting solid tumors are now also being explored in preclinical settings. For this, NK cell-specific co-stimulatory domains are being explored to replace T-cell specific domains in an attempt to increment NK cell-specific activation. Two promising approaches target the prostate stem cell antigen (PSCA), highly expressed on primary prostate tumors and metastases (73), and the epidermal growth factor type III (EGFRvIII), expressed on glioblastoma cells, using the DNAX-activation protein (DAP)12 stimulatory domain instead of one of the commonplace T-cell domains like CD3ζ (74, 75). DAP12 expression in NK cells induces NKG2C and NKp44 expression upon stimulation. Another group generated an NKG2D-DAP10-CD3ζ construct (49). The DAP10 domain induces NK cell activation upon phosphorylation through NKG2D-mediated ligand binding (76, 77). Although NKG2D is technically not an scFv, the construct is referred to as a CAR, because of its receptor structure. Recently, the NKG2D transmembrane domain has been combined with an anti-mesothelin scFv and the NK cell-specific signaling domain 2B4 and CD3ζ to target ovarian cancers (62). This construct, cloned in a PiggyBac transposon system, was expressed on iPSC-NK cells, and showed improved specificity and cytotoxicity against the mesothelin-expressing ovarian cancer cell line A1847 and in an ovarian cancer xenograft mouse model. In this mouse model, CAR-iPSC-NK activity was compared with an anti-mesothelin CAR-T expressed in primary T cells and showed similar anti-tumor activity but significantly lower toxicity and prolonged survival. Of note is also a platform that combines NK cell and T cell advantages, by expressing TCR-CAR chimeric constructs on the NK cell surface (78). Recently, the biotech companies Glycostem Therapeutics and Zelluna Immunotherapies announced a partnership to further develop this field.

**Table 3:** Ongoing clinical trials with CAR-NK cells.

CAR Target	Condition	Study Phase	NCT
**CD19**	B-Lymphoid Malignancies/ALL/CLL	I/II	NCT03056339
**CD19/CD22**	B-Cell Lymphoma	I	NCT03824964
**CD19**	B-Cell Lymphoma	I	NCT03690310
**CD19**	ALL/CLL	I/II	NCT02892695
**CD19**	ALL	I	NCT00995137
**CD33**	AML	I/II	NCT02944162
**CD7**	AML/T-cell leukemia	I/II	NCT02742727
**CD22**	B-Cell Lymphoma	I	NCT03692767
**BCMA**	MM	I/II	NCT03940833
**HER-2**	Glioblastoma	II	NCT03383978
**Mesothelin**	Ovarian cancer	I	NCT03692637
**PSMA**	Prostate cancer	I	NCT03692663
**ROBO1**	Pancreatic cancer	I/II	NCT03941457
**ROBO1**	Solid Tumors	I/II	NCT03931720
**ROBO1**	Solid Tumors	I/II	NCT03940820
**NKG2DL**	Solid Tumors	I	NCT03415100
**MUC1**	Solid Tumors	I	NCT02839954
**N.A.**	NSCLC	I	NCT03656705
**ACE2/NKG2DL**	COVID-19	I/II	NCT04324996

ALL, Acute Lymphocytic Leukemia; AML, Acute Myeloid Leukemia; CLL, Chronic Lymphocytic Leukemia; MM, Multiple Myeloma; NSCLC, Non-small Cell Lung Cancer.

### NK Cells With Downregulated Inhibitory Receptors

Cancer cells can throttle immune responses by stimulating key regulators on the surface of immune effector cells known as inhibitory checkpoint molecules. Identification and targeting of inhibitory checkpoints significantly boost immune responses and is therefore of major interest in cancer immunotherapy. During *ex vivo* expansion of NK cells, some inhibitory receptors, like NKG2A, are still highly expressed (79–81), suggesting a critical role in NK cell maturation. On the other end, inhibitory receptors curb NK cell cytotoxic activity and reduce therapeutic efficacy in clinical settings. Indeed, hyporesponsive NKG2A-expressing NK cells are prominent within the TME, thus stressing the importance of this receptor in reducing NK cells activity (82). NKG2A dimerizes with CD94 to bind HLA-E molecules loaded with tumor peptides. While HLA-E surface expression in tumor cells is very weak, IFN-γ produced by NK cells can cause its overexpression (29, 50, 83). Once the peptide/HLA-E complex is stabilized and binds NKG2A, NK cell activity is dampened. In contrast, RNAi-mediated inhibition of NKG2A expression by shRNA improved NK cell *in vitro* activity against an HLA-E expressing B-lymphoblastoid cell line (53). Notably, NK cell cytotoxicity was also enhanced against the AML-derived, HLA-E-negative cell line K562. This increased HLA-E independent cytotoxicity was probably caused by increased activating NKp30 receptor levels in the NKG2A-negative cell. Unfortunately, these cells have only been tested on a small set of HLA-E positive/negative cell lines. Another group downregulated NKG2A function in PBNKs and NK-92 cells by linking an anti-NKG2A antibody to an endoplasmic reticulum-retention domain, and achieved increased cytotoxicity against both HLA-E-positive and -negative cells derived from Ewing’s sarcoma, osteosarcoma and AML, as well as prolonged survival in immunodeficient mice expressing HLA-E tumors (50). Blocking of inhibitory receptors represents a feasible approach within the field of biotech industry, although the studies focused on NKG2A only, and their number so far is very limited. Additionally, data comparing NKG2A-negative NK cells and CAR-NK cells activity against the same tumor type are lacking, precluding a comparison between both strategies.

### NK Cells With Modified ADCC

FcγRIII (CD16)-mediated antibody-dependent cell-mediated cytotoxicity (ADCC) plays an important role in tumor clearance. The CD16 isoform expressed on NK cells (CD16a) has two allelic variants with a phenylalanine (F) or valine (V) at amino acid 158, resulting in low (CD16a-158-F/F) and high affinity (CD16a-158-V/V) Fc receptor isoforms. Several studies showed that patients that express the CD16a-F/F variant have better therapy response than patients that are either heterozygous (CD16a-158-V/F) or homozygous for V upon mAb treatment (84, 85). CD16a expression is downregulated upon NK cell activation, mainly due to matrix metalloproteases shedding (86). NK-92 cells, an attractive and cheap source for clinical applications of NK cells, cannot mediate ADCC as they lack CD16 expression. In an attempt to constitutively enhance ADCC activity of NK-92 cells, these cells have been modified to express a CD16a chimera fused to the CD28 and 4-1BB co-stimulatory domains. Expression of the construct enhanced their cytotoxic activity and restored ADCC activity against CD20-positive tumor cells (87). In another attempt, ADCC potency of NK-92 cells was improved by transduction of the high-affinity CD16a-V158 mutant that is resistant to ADAM17-mediated cleavage and shedding (88, 89). This cell line, (haNK, developed by NantKwest), showed improved killing capacity compared to PBNK cells from healthy donors and is now in clinical trials against breast cancer (NCT03387085), Merkel cell carcinoma (NCT03853317) and squamous cell carcinoma (NCT03387111). CD16a-158V expression in NK-92 and iPSCs-derived NK cells led to enhanced NK cell activation in the presence of rituximab (90), as well as in PBNKs, although overexpression was transient and lasted no more than 3 days (60). CRISPR/Cas9 has been applied in NK-92 cells by nucleofection to restore endogenous CD16 and DNAM-1 expression by introducing a new promoter upstream the endogenous genes (61). Although this approach was successful, the low nucleofection efficiency and its very high toll on NK cell viability currently preclude its application in *ex vivo* expanded NK cells, limiting it to NK cell lines capable of autonomous and indefinite growth. As CD16 overexpression markedly improved NK cell activity and cytokine secretion, combinatorial approaches are currently under investigation. For example, NantKwest is employing CAR technology on haNK cells, and has also initiated a phase I clinical study against PD-L1 expressing non-Small Cell Lung Cancer.

### NK Cells With Increased Persistence and Proliferation Potential

*In vivo* persistence of NK cells strictly depends on exogenous cytokines. Allogeneic NK cell survival is typically restricted to a couple of weeks, necessitating multiple infusions to achieve therapeutic effects (40). Administration of recombinant IL-2 (rIL-2) in clinical settings resulted in severe toxicities at high doses and activation of inhibitory T-regs at low doses (91). To circumvent these drawbacks, NK-92 cells have been transduced with the IL-2 gene, abolishing NK-92 growth dependence on exogenous IL-2 (92). Transduced NK-92 cells have been tested in nude mice with 3-day-established liver metastases, without exerting side effects after 6 months of treatment. With the discovery that IL-15 has higher potency and lower toxicity than IL-2 (93), the focus has shifted to this cytokine. IL-15 exists in both a soluble and membrane-bound isoforms complexed with IL-15 Rα (94, 95). NK cells growth dependence on IL-15 has been circumvented by retrovirally transducing PBNK cells with the mbIL-15 membrane-bound isoform, increasing *in vivo* NK cell persistence without the need for exogenous IL-2 or IL-15 (46). Stimulatory cytokine signaling has also been recently combined with CAR-NK technology. Briefly, CB-derived NK cells were transduced with a retrovirus encoding a CD19 CAR, soluble IL-15 and the iC9 suicide gene (96). These NK cells had prolonged *in vivo* survival and were able to control tumor progression significantly better than non-modified or CD19 CAR-only NK cells. The MD Anderson Cancer Centre phase I clinical study followed, as mentioned above. A CAR construct, targeting the EpCAM carcinoma antigen epithelial cell adhesion molecule, that also encoded IL-15 has been transduced into NK-92 cells (56). The IL-15 transgene induced strong proliferation signals, allowing transduced NK-92 cells to grow in the absence of stimulatory cytokines and, additionally, acting as a selection marker for the transduced cells. The CAR gene selectively improved NK cell cytotoxicity against EpCAM-expressing cell lines.

NK cells have low proliferative potential, possibly because of progressive telomere shortening during division cycles. The process can be partially overcome by overexpressing the telomerase reverse transcriptase protein (TERT) responsible for telomere end restoration, normally very lowly expressed in primary cells. Indeed, NK-cell lifespan has already been extended to several months by hTERT overexpression in PBNKs (47). However, transduced PBNKs were still not capable of autonomous growth and proliferation remained dependent on cell-to-cell contacts with feeder cells. Such “immortalized” NK cells can be useful for clinical applications, where large amounts of NK cells need to be injected, and donor availability and/or variability is limiting. Nevertheless, the use of feeder cells in clinical manufacture is not accepted by all regulatory authorities, as it might pose safety concerns for patients. Consequently, feeder cell-free culture systems need to be developed for worldwide implementation. Also, hTERT itself can pose safety concerns, as it might drive uncontrolled proliferation, and might require failsafe mechanisms like a suicide gene.

### NK Cells With Increased Tumor Homing

Immune cell homing to and infiltration of tumors is a fundamental prerequisite for effective tumor killing. NK cell ability to infiltrate into the tumor stroma is limited, negatively affecting NK cell therapy efficacy (97). The chemokine receptor CCR7 redirects NK cells preferentially to lymph node-associated chemokine CCL19. CCR7 mRNA transfection into PBNKs by mRNA electroporation improved their *in vitro* migration towards CCL19 (60). Another group used primary NK cells transduced with retrovirus encoding CXCR2 to improve trafficking towards renal cell carcinoma (48). The NK-like YT cell line was transduced with lentivirus encoding an anti-EGFRvIII CAR and the chemokine receptor CXCR4 (75). The CXCR4 receptor promoted specific chemotaxis to glioblastoma cells secreting the CXCL12/SDF-1α chemokine, while the αEGFRvIII-CAR improved the killing specificity and cytotoxicity. The approach also increased tumor regression and survival in xenograft mouse models.

These studies, although limited and mainly *in vitro*, demonstrate that NK cell homing to specific tumor sites can improve NK cell-mediated tumor clearance, especially if combined with strategies to enhance NK cell functions after migration to the tumor site.

### NK Cells With Increased Resistance to the Tumor Microenvironment

One of the major immunosuppressive factors within the TME is TGF-β, produced by various stromal cells, T-regs, MDSCs, and the tumor cells. TGF-β interferes with NK cell activation by counteracting several important activating receptors. It downregulates NK cell NKG2D and NKp30 surface expression (98, 99) and inhibits CD16-mediated IFN-γ production and ADCC *in vitro (* 100). RNAi-mediated knockdown of SMAD3, a TGF-β receptor signal transducer, by transduction of NK-92 cells with a lentivirus encoding SMAD3 shRNA increased IFN-γ, perforin and granzyme B expression, and enhanced cytotoxicity, increasing tolerance to TGF-β signaling both *in vitro* and *in vivo (* 101). Expression of a chimera consisting of the TGF-β receptor type II (TGFBR2) extracellular and transmembrane domain fused to the NKG2D intracellular domain on NK-92 cells caused tolerance to the TGF-β signaling and improved chemoattraction to TGF-β-secreting tumor cell lines (102). Additionally, these NK-92 cells inhibited naïve CD4^+^ T cell to T-reg differentiation by IFN-γ signaling. A dominant-negative mutant form of TGFBR2 (DNRII) has been expressed in CB-derived NK cells to block or decrease TGF-β signaling. These NK cells did not show downregulation of activating receptors NKG2D and DNAM-1 or of granzyme B and perforin upon TGF-β stimulation (51). The same group further created an improved version of the DNRII receptor (renamed “NKA” receptor) by fusing its extracellular domain to DAP12, providing NK cell activating signals upon TGF-β stimulation. CB-derived NK cells expressing NKA had enhanced cell cytotoxic activity and persistence against neuroblastoma both *in vitro* and *in vivo (* 52). A similar approach with the DNRTβII receptor in NK-92 cells increased their resistance to TGF-β signaling, potentiating antitumor activity in an *in vivo* lung cancer murine model (63).

Adenosine is emerging as another key negative regulator of NK cells within the TME. Adenosine signals via the A_2A_R receptor and limits NK cell maturation, negatively affecting their proliferation and tumor control (35). Blockage of A_2A_R with an inhibitor caused anti-metastatic effects in breast cancer and melanoma mouse models (103). mAb-mediated inhibition of CD73 ectonucleotidase, one of the key enzymes responsible for extracellular adenosine synthesis, in combination with NKG2D CAR NK-92 cells generated using a PiggyBac transposon system, improved control of CD73-positive tumors (64). The combinatorial approach has been tested against cell lines of prostate cancer (PC3), lung carcinoma (A549) and glioblastoma (GBM43 and GBM10) expressing high levels of CD73 and a xenograft mouse model of lung carcinoma with A549 cells. Although the mAb-CAR combined approach did not dramatically improve anti-tumor activity of the NK-92 cells, the study is important as a first demonstration that stable CAR integration with a non-viral system in NK cells is feasible, and can be extended to other constructs.

## Future Perspectives and Concluding Remarks

NK cell trials have been ongoing for several years by now, demonstrating the safety and efficacy of NK cell-based immunotherapies, especially against hematological malignancies. So far, efficacy against solid tumors is limited, and requires additional technology. NK cells were quite refractory to standard genetic manipulation techniques, resulting in major delays of the first clinical trials with genetically engineered NK cell. Substantial improvements in the last years has led to the first trials. Nonetheless, challenges remain. Viral-based genetic manipulation of NK cells is currently the gold standard to stably express exogenous genes, but transduction efficiency and transgene expression levels are still variable, requiring multiple transductions rounds or post-transduction enrichment. Besides, insertional mutagenesis needs continued safety monitoring. Non-viral delivery methods are still far from routine implementation, as the efficiency and viability can be very low, and the transgene expression can be transient. Low efficiencies are not a great disadvantage with immortalized cells like NK-92, as these can be enriched and then grown indefinitely. But *ex vivo* expanded NK cells have a limited proliferation potential and enter senescence relatively soon (47). For these cells, highly effective engineering strategies are much more important.

As mentioned above, NK cell efficacy against solid tumors is limited compared to hematological malignancies. Introduction of CAR constructs into NK cells, restoring ADCC functions and/or downregulation of inhibitory receptors can dramatically potentiate their effector functions, helping the patient’s immune system in eradicating the disease. The need for NK-specific CAR constructs is now widely recognized, as T-cell-based CARs have reduced activation potentials compared to the former ones, and high-throughput screening techniques will be essential for their identification (66). The limited proliferation potential of NK cells usually does not allow them to persist longer than 2–3 weeks after injection *in vivo*. Furthermore, homing to tumor sites is often hampered by the TME. Consequently, a highly cytotoxic potential could be relatively limited if NK cells do not persist long enough to eradicate malignant cells or home in on tumor sites. Early efforts in providing NK cells with stimulatory cytokines prolonging half-life like IL-15 are encouraging and worth being further developed, especially in combination with CARs or other activating receptors. Similarly, increasing expression of chemokine receptors on the NK cell surface improves NK cells targeted trafficking and tumor eradication, paving the way for combinatorial strategies. Many solid tumors are difficult to target also because they are encapsulated and protected by a thick layer of extracellular matrix (ECM), a mesh made up mainly by insoluble proteins like type IV collagen and heparan sulphate proteoglycans (HSPGs), that reduce the infiltration abilities of NK cells (104). Heparanase is upregulated in activated NK cells, improving migration within tumor stroma and playing an important role in reducing tumor growth and metastases (104). To conclude, understanding NK cell biology is another key factor that will help to improve genetic engineering strategies and overcome tumor resistance mechanisms, and allow to fully unleash anti-cancer NK cell potential.

## Author Contributions

SM wrote the paper. LK, DG, and JS reviewed the paper. All authors contributed to the article and approved the submitted version.

## Funding

This work was supported by a network grant of the European Commission (H2020-MSC-ITN-765104-MATURE-NK) to Glycostem Therapeutics and SM was a fellow in the project, and by a joint grant of the European Commission and EUREKA (E!11764 MODIFY-NK) to Glycostem Therapeutics.

## Conflict of Interest

SM, DG, JP and LK are employed by Glycostem Therapeutics.
